# The burden of Gastric Cancer and possible risk factors from 1990 to 2021, and projections until 2035: findings from the Global Burden of Disease Study 2021

**DOI:** 10.1186/s40364-024-00720-8

**Published:** 2025-01-07

**Authors:** Niping Qin, Yangyan Fan, Tao Yang, Zhiping Yang, Daiming Fan

**Affiliations:** 1 First Hospital of Shanxi Medical University, Scholl of Management of Shanxi Medical University, Taiyuan, 030001 China; 2 The First Affiliated Hospital, Guizhou University of Traditional Chinese Medicine, Guiyang, 550001 China; 3 State Key Laboratory of Holistic Integrative Management of Gastrointestinal Cancers and Xijing Hospital of Digestive Diseases, Fourth Military Medical University, No. 127 Changle West Road, Xi’an, 710032 China

**Keywords:** Gastric cancer, Global burden of disease, Risk factors, Mortality, Disability-adjusted life years

## Abstract

**Background and objective:**

Gastric cancer (GC) remains a significant global health challenge, characterized by high incidence and mortality rates, particularly in East Asia. A comprehensive understanding of the disease burden of gastric cancer is crucial for developing effective prevention and treatment strategies. However, comprehensive global assessments of the disease burden of gastric cancer remain limited. This study, based on the Global Burden of Disease (GBD) framework, systematically analyzes global trends in gastric cancer from 1990 to 2021 and projects future trends through 2035, aiming to provide scientific evidence for policymaking.

**Methods:**

The data were derived from the Global Burden of Disease (GBD) Study 2021, covering gastric cancer (GC) incidence, mortality, disability-adjusted life years (DALYs), age-standardized incidence rates (ASIRs), age-standardized death rates (ASDRs), and age-standardized DALY rates (ASRs) across 204 countries and regions from 1990 to 2021. The Bayesian age-period-cohort model was employed to project trends up to 2035.

**Results:**

In comparison with 1990, both the incidence and mortality of GC rose in 2021, with over 1.23 million new cases recorded globally, resulting in 954,373.60 deaths and 22,786,633.10 DALYs. Between 1990 and 2021, the ASIRs, ASDRs, and ASRs decreased by 42% (ranging from 49 to 35%), 49% (ranging from 55 to 43%), and 53% (ranging from 58 to 47%), respectively. The peak ASIRs and ASDRs in 2021 were seen in the high-middle SDI quintile. Males exhibited higher rates of ASDRs, ASIRs, and ASRs compared to females. In 2021, East Asia and high-income North America bore the largest burden of smoking-related GC, while Central Europe experienced the highest burden from high-sodium diets. Forecasts toward 2035 indicate a continued decline in both ASIRs and ASDRs.

**Conclusions:**

Despite notable reductions in both incidence and mortality, GC remains a substantial global burden, affecting various regions and countries. Deaths and DALYs related to high-sodium diets and smoking have shown an overall decline. However, substantial regional and age-related disparities persist. Targeted interventions, such as smoking control and promoting the intake of fresh fruits and vegetables, are essential in diminishing GC risk.

**Supplementary Information:**

The online version contains supplementary material available at 10.1186/s40364-024-00720-8.

## Introduction

Gastric cancer (GC) exists as a leading malignant condition across the globe [1]. Based on data from the GLOBOCAN initiative by the International Agency for Research on Cancer, it emerged as the fifth most prevalent malignancy in 2018, responsible for 8.2% of all cancer-related fatalities [2]. Being a malignant tumor, GC imposes a substantial disease burden [3]. Despite decreasing trends in occurrence and mortality rates (MRs) throughout recent decades, roughly 1.089 million new diagnoses and 0.769 million fatalities were documented globally in 2020 [4]. The occurrence and MRs of GC exhibit considerable variation across different regions, with a notable decline in developed nations in recent years [5]. In contrast, East Asia, characterized by its large population, has historically borne the highest incidence of GC, although a similar downward trend has been recorded. In 2018, GC mortality reached its peak in East Asia (15.9 per 100,000 individuals) while remaining lowest in North America (1.8 per 100,000 individuals) [6].

Appropriate preventive protocols must be implemented, as prevention represents a critical strategy for controlling GC and reducing its MR [7]. Effective prevention relies on accurately determining the contributing risk factors (RFs) linked to GC [8]. Research has demonstrated that GC emerges from multiple causes, incorporating factors such as Helicobacter pylori (Hp) colonization, surrounding conditions, inherited predispositions, and personal habits [9]. Daily lifestyle selections, particularly excessive salt consumption and tobacco use, exhibit substantial effects on cancer outcomes throughout an individual’s lifespan [10, 11]. A meta-analysis of case-control investigations has demonstrated a notable connection linking elevated sodium consumption and GC occurrence, where increased salt consumption exhibited greater risk levels versus minimal intake (*OR* = 1.55, 95% *CI* [1.45, 1.64]; *p* < 0.001) [12].

The impact of GC varies notably among regions, encompassing geographical location, dietary patterns, and the level of medical advancement. Research has indicated that East Asian nations (e.g., China, Japan, and South Korea) exhibit notably elevated incidence and MRs for GC compared to Western countries, encompassing the United States [13]. These East Asian countries are responsible for approximately two-thirds of global GC cases, primarily attributed to high Hp infection prevalence and distinctive food consumption patterns, alongside additional RFs [14]. Consequently, examining the health impact, RFs, and chronological patterns of GC across various countries and regions provides decision-makers with a comprehensive understanding of the global epidemic, offering valuable insights for developing targeted and successful preventive measures and management approaches.

Although earlier research has offered calculations of the GC disease burden, these have typically been restricted to specific territories or nations with sparse data, particularly those in East Asia. Contemporary statistics regarding worldwide impact and patterns associated with GC occurrence and MRs remain inadequately explored. While recent research has explored the burden of GC among adolescents and young adults, a thorough evaluation of this burden across the global population and various regions has yet to be conducted.

This study sought to examine the impact, patterns, and potential determinants linked to GC across various nations and territories from 1990 to 2021, utilizing data from the Global Burden of Disease (GBD) Study 2021 and to project the cancer burden through 2030. A comprehensive grasp of current burden distributions is essential for aiding policymakers in more effectively allocating health resources and alleviating the impact of GC.

## Methodologies and materials

### Data sources

Data on GC from the 1990–2021 period were obtained through the Global Health Data Exchange (GHDx) query tool. The investigation incorporated information about mortality figures, occurrence rates, and disability-adjusted life-years (DALYs); age-standardized death rate (ASDR), age-standardized incidence rate (ASIR); age-standardized DALYs rate (ASR) as well as potential RFs for GC were sourced from the GBD 2021. The research methodology used by the GBD has been extensively described in the existing literature. The socio-demographic index (SDI), a comprehensive metric, integrates per capita lag-distributed income with educational attainment among individuals above 15 years to represent regional economic conditions [15]. The SDI utilizes a scale from 0 to 1, where elevated values denote enhanced socioeconomic progress. Regional classifications based on SDI comprise five cohorts: low (< 0.46), low-middle (0.46–0.60), middle (0.61–0.69), high-middle (0.70–0.81), and high (> 0.81) [16]. Furthermore, projections of the ASDR and ASIR for GC across different age cohorts are made for the 2022–2032 period.

### Statistical analysis

To normalize differences in population age structures, calculations of ASDR, ASIR, and ASR were employed to evaluate GC impact and patterns across different areas, utilizing the GBD Standard Population Distribution [17]. Research indicates that the Bayesian age-period-cohort (BAPC) framework delivers enhanced precision in predicting cancer statistics when compared to alternative methods like generalized additive, joinpoint, and Poisson regression analyses [18]. In this investigation, projections of GC-related ASIR and ASDR for 2022–2032 were generated using the BAPC methodology integrated with nested Laplace approximation techniques.

## Results

### GC burden in 2021

In 2021, the global number of GC-related deaths was 954,373.60, while the incidence exceeded 1.23 million cases. The ASDR for GC was 11.2 per 100,000 person-years, reflecting a decrease of 49% (-55% to -43%) since 1990. Similarly, the ASIR was 14.33 per 100,000 person-years, showing a decline of 42% (-49% to -35%) from 1990. GC was responsible for 22,786,633.10 DALYs worldwide in 2021. The ASR stood at 262.75, representing a 53% (-58% to -47%) reduction from 1990 to 2021 (Table 1).

**Table 1:** Incidence, deaths and DALYs of gastric cancer in 2021, and percentage change of age-standardized rates by GBD region

World region	Deaths			Incidence			DALYs		
	ASDR per 100 000	Percentage change in rates, between1990 and 2021	Percentage change in rates, between2019 and 2021	ASIR per 100,000	Percentage change in rates, between 1990 and 2021	Percentage change in rates, between2019 and 2022	ASR per 100,000	Percentage change in rates, between 1990 and 2021	Percentage change in rates, between2019 and 2021
Western Sub-Saharan Africa	6.69	-0.16(-0.30, -0.02)	-3.29 (-2.89, -3.64)	6.33	-0.16(-0.31, -0.02)	-3.29 (-2.89 - -3.64)	156.08	-0.21(-0.35, -0.06)	-2.73 (-3.15, -2.21)
Western Europe	5.57	-0.62(-0.64, -0.60)	-2.84 (-3.17, -2.52)	7.81	-0.54(-0.56, -0.52)	-2.84 (-3.17 - -2.52)	120.38	-0.62(-0.64, -0.60)	-3.97 (-4.28, -3.48)
Tropical Latin America	9.78	-0.50(-0.52, -0.48)	-0.37 (-1.41, 0.74)	9.99	-0.47(-0.50, -0.45)	-0.37 (-1.41, − 0.74)	237.81	-0.49(-0.51, -0.46)	0.69 (0.03, 1.69)
Southern Sub-Saharan Africa	7.13	-0.12(-0.22, -0.01)	1.52 (2.05, 1.49)	7.02	-0.11(-0.21, -0.00)	1.52 (2.05, 1.49)	177.66	-0.15(-0.25, -0.03)	2.60 (4.41, 2.74)
Southern Latin America	10.51	-0.44(-0.49, -0.38)	-7.02 (-7.62, -6.38)	11.39	-0.39(-0.44, -0.33)	-7.02 (-7.62, -6.38)	241.14	-0.44(-0.49, -0.39)	-6.92 (-7.86, -6.14)
Southeast Asia	6.83	-0.37(-0.45, -0.24)	2.20 (3.81, -0.03)	7.27	-0.32(-0.41, -0.18)	2.20 (3.81, -0.03)	170.78	-0.40(-0.48, -0.27)	1.19 (2.89, -0.23)
South Asia	5.72	-0.27(-0.37, -0.15)	-1.50 (-2.83, -2.57)	5.7	-0.26(-0.36, -0.13)	-1.50 (-2.83, -2.57)	145.55	-0.32(-0.41, -0.21)	-1.43 (-1.58, -2.71)
Oceania	13.45	-0.24(-0.40, -0.04)	-0.55 (-0.70, 0.32)	13.26	-0.23(-0.39, -0.02)	-0.55 (-0.70, − 0.32)	341.81	-0.25(-0.43, -0.03)	-0.80 (-1.53, 0.34)
North Africa and Middle East	9.43	-0.38(-0.45, -0.29)	-1.23 (-0.17, 0.93)	9.67	-0.34(-0.42, -0.25)	-1.23 (-0.17, − 0.93)	217.41	-0.43(-0.50, -0.35)	-0.79 (-1.44, 1.88)
Middle SDI	13.72	-0.51(-0.59, -0.42)	1.45 (0.07, 2.03)	16.91	-0.41(-0.51, -0.29)	1.45 (0.07, 2.03)	320.24	-0.56(-0.63, -0.46)	0.49 (-0.19, 1.13)
Low-middle SDI	7.71	-0.26(-0.34, -0.17)	-1.63 (-0.54, -3.99)	7.68	-0.24(-0.32, -0.14)	-1.63 (-0.54, -3.99)	192.56	-0.30(-0.37, -0.21)	-1.44 (0.12, -4.89)
Low SDI	8.46	-0.29(-0.36, -0.19)	-2.41 (-3.52, -2.10)	8.13	-0.29(-0.36, -0.19)	-2.41 (-3.52, -2.10)	209.77	-0.33(-0.40, -0.23)	-1.49 (-2.56, -1.09)
High-middle SDI	14.93	-0.52(-0.59, -0.44)	1.53 (1.55, 3.22)	19.62	-0.41(-0.50, -0.30)	1.53 (1.55, 3.22)	353.18	-0.56(-0.63, -0.47)	0.60 (-0.31, 2.57)
High-income North America	2.95	-0.47(-0.49, -0.45)	0.90 (1.03, 1.20)	5.23	-0.37(-0.39, -0.35)	0.90 (1.03, 1.20)	70.88	-0.45(-0.47, -0.44)	-0.06 (-0.03, 0.28)
High-income Asia Pacific	13.13	-0.65(-0.67, -0.62)	3.00 (2.53, 3.40)	25.43	-0.61(-0.64, -0.58)	3.00 (2.53, 3.40)	273.07	-0.70(-0.72, -0.67)	1.05 (0.46, 1.40)
High SDI	6.83	-0.57(-0.59, -0.55)	0.59 (0.51, 1.05)	11.16	-0.52(-0.54, -0.49)	0.59 (0.51, 1.05)	146.1	-0.62(-0.64, -0.59)	-0.84 (-1.64, -0.55)
global	11.2	-0.49(-0.55, -0.43)	0.25 (0.35, 1.64)	14.33	-0.42(-0.49, -0.35)	0.25 (0.35, 1.64)	262.75	-0.53(-0.58, -0.47)	-0.49 (-0.45, 0.66)
Eastern Sub-Saharan Africa	7.09	-0.36(-0.44, -0.25)	-1.07 (-2.87, 0.21)	6.8	-0.36(-0.44, -0.25)	-1.07 (-2.87, − 0.21)	175.48	-0.40(-0.48, -0.29)	-0.46 (-2.93, 0.39)
Eastern Europe	12.15	-0.59(-0.62, -0.56)	-1.44 (-4.88, 1.35)	14.67	-0.55(-0.59, -0.52)	-1.44 (-4.88, − 1.35)	306.55	-0.62(-0.65, -0.59)	-1.68 (-6.15, 1.62)
East Asia	21.26	-0.53(-0.62, -0.40)	3.14 (3.54, 4.12)	28.64	-0.39(-0.52, -0.21)	3.14 (3.54, 4.12)	496.94	-0.57(-0.66, -0.44)	1.73 (1.33, 1.76)
Central Sub-Saharan Africa	8.93	-0.26(-0.42, -0.05)	-0.46 (-0.40, -0.62)	8.54	-0.26(-0.42, -0.05)	-0.46 (-0.40, -0.62)	220.04	-0.28(-0.45, -0.06)	-0.12 (0.06, -0.23)
Central Latin America	11.69	-0.45(-0.50, -0.38)	3.19 (-2.66, 10.09)	11.98	-0.41(-0.47, -0.34)	3.19 (-2.66, 10.09)	282.56	-0.41(-0.48, -0.34)	3.29 (-3.26, 10.81)
Central Europe	8.54	-0.54(-0.57, -0.50)	-0.43 (-3.06, 2.38)	9.19	-0.50(-0.54, -0.46)	-0.43 (-3.06, 2.38)	201.3	-0.55(-0.58, -0.51)	-1.33 (-4.39, 2.14)
Central Asia	11.56	-0.56(-0.60, -0.51)	-2.35 (-5.69, 0.11)	11.78	-0.55(-0.60, -0.51)	-2.35 (-5.69, 0.11)	298.62	-0.59(-0.63, -0.54)	-2.51 (-6.14, 0.03)
Caribbean	7.79	-0.37(-0.45, -0.28)	-0.38 (-1.95, 1.40)	7.97	-0.34(-0.43, -0.25)	-0.38 (-1.95, 1.40)	195.67	-0.34(-0.44, -0.23)	-1.01 (-2.00, 0.05)
Australasia	3.91	-0.49(-0.54, -0.45)	-1.44 (-2.86, -1.14)	5.93	-0.42(-0.47, -0.37)	-1.44 (-2.86, -1.14)	84.88	-0.51(-0.55, -0.46)	-2.76 (-3.38, -2.08)
Andean Latin America	21.33	-0.35(-0.47, -0.19)	-7.46 (-11.00, -2.82)	21.48	-0.31(-0.44, -0.13)	-7.46 (-11.00, -2.82)	485.41	-0.37(-0.50, -0.21)	-5.84 (-9.67, -0.39)

ASDR, the age-standardized death rate. ASIR, the age-standardized incidence rate. ASR, the age-standardized DALYs rate

In 2021, the highest and lowest ASDRs for GC were recorded in the high-middle SDI quintile (14.93 per 100,000 person-years) and the high SDI quintile (6.83), respectively. Similarly, the highest and lowest ASIRs for GC were observed in the high-middle SDI quintile (19.62) and the low-middle SDI quintile (7.68), respectively.

On a regional basis, the highest ASDR per 100,000 person-years in 2021 was found in Andean Latin America (21.33), with East Asia (21.26) following closely behind. In contrast, high-income North America exhibited the smallest rate (2.95). The top five regions with the highest ASIRs per 100,000 person-years were East Asia (28.64), high-income Asia Pacific (25.43), Andean Latin America (21.48), Eastern Europe (14.67), and Oceania (13.26). Notable variations emerged across regions in age-standardized rates (ASRs). In 2021, the highest ASRs per 100,000 person-years were observed in East Asia (496.94), succeeded by Andean Latin America (485.41) and Oceania (341.81), while Australasia (84.88) and high-income North America (70.88) reported the lowest values.

**Fig. 1:**
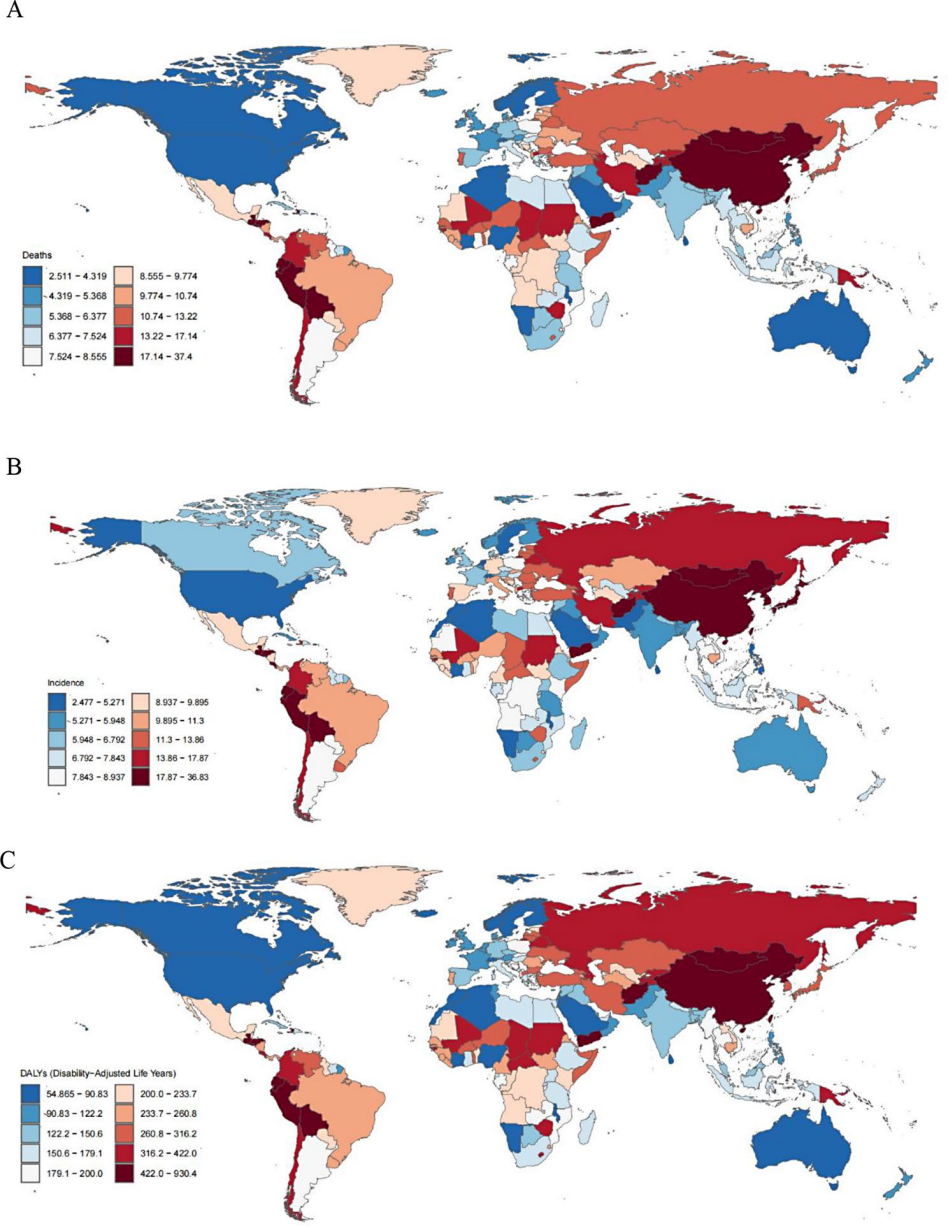
Age-standardized rates for deaths (**A**), incidence (**B**) and DALYs (**C**) of gastric cancer across 204 countries and territories, 2021. DALYs, disability-adjusted life-years

**Fig. 2:**
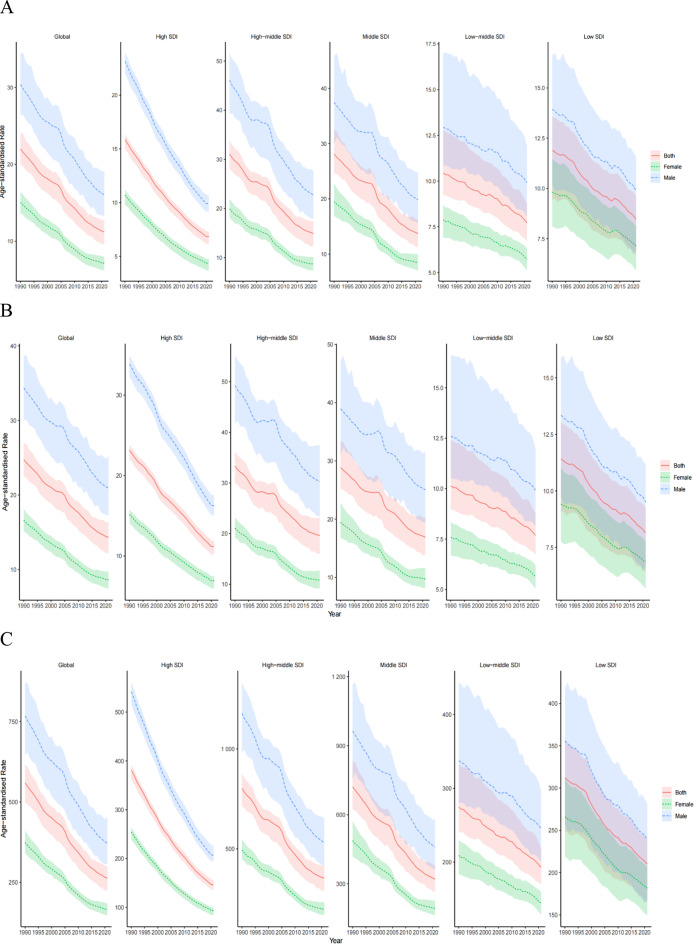
Time of trend of the age-standardized death, incidence and DALYs rate of gastric cancer by sex in 1990–2021. **A**. The ASDR of gastric cancer. **B**. The ASIR of stomach cancer. **C**. The age-standardized DALYs rate of stomach cancer

Figure 1 illustrates the global distribution of ASDRs, ASIRs, and ASRs for GC in 2021. The nations with the highest ASDRs were Peru, Bolivia, and Ecuador from Andean Latin America, along with China and Mongolia from East Asia, whereas the minimal ASDRs were recorded in the United States, Canada, Australia, Algeria, and Saudi Arabia (Fig. 1A). The United States stood among the territories exhibiting reduced ASIR. In East Asia, elevated ASIRs were documented in China, Mongolia, Japan, and South Korea (Fig. 1B). In 2021, the ASDRs, ASIRs, and ASRs were greater in males than in females (Fig. 2).

A notable global trend was observed in GC epidemiology during the COVID-19 pandemic. From 2019 to 2021, Both the GC mortality (0.25, 95% CI: 0.35–1.64) and incidence (0.25, 95% CI: 0.35–1.64) rates increased, while DALYs declined (-0.49, 95% CI: -0.45–0.66). Notably, in high-middle and middle SDI regions, the occurrence, mortality, and DALY rates increased more markedly than the global average. By contrast, low-SDI and low-middle SDI regions exhibited a continued downward trend in incidence, mortality, and DALY rates, remaining below the global average. This trend was particularly pronounced in high-income Asia Pacific, East Asia, and Central Latin America, where both the GC incidence and MRs exhibited significant increases. Furthermore, the DALYs rate exhibited the most prominent increase in Central Latin America and Southern Sub-Saharan Africa (Table 1).

### Time trends of GC disease burden in regions with diverse SDI levels from 1990 to 2021

From 1990 to 2021, global reductions were observed in the ASDRs, ASIRs, and ASRs of GC (Table 1; Fig. 2), exhibiting a steeper decline among males versus females. Significant reductions in the ASDRs, ASIRs, and ASRs were reported across all SDI regions throughout this timeframe. The most pronounced decreases were recorded in the high-middle SDI regions, succeeded by the middle and high SDI regions (Fig. 2). In high-SDI, low-middle SDI, and low-SDI regions, the ASDRs were below the global average (Fig. 2A). Conversely, the ASIRs in high-middle and middle SDI regions exceeded the global average (Fig. 2B). Furthermore, ASRs in high-middle and middle SDI regions surpassed the worldwide average, whereas those in the high SDI, low-middle SDI, and low SDI regions were below the worldwide average (Fig. 2C).

**Fig. 3:**
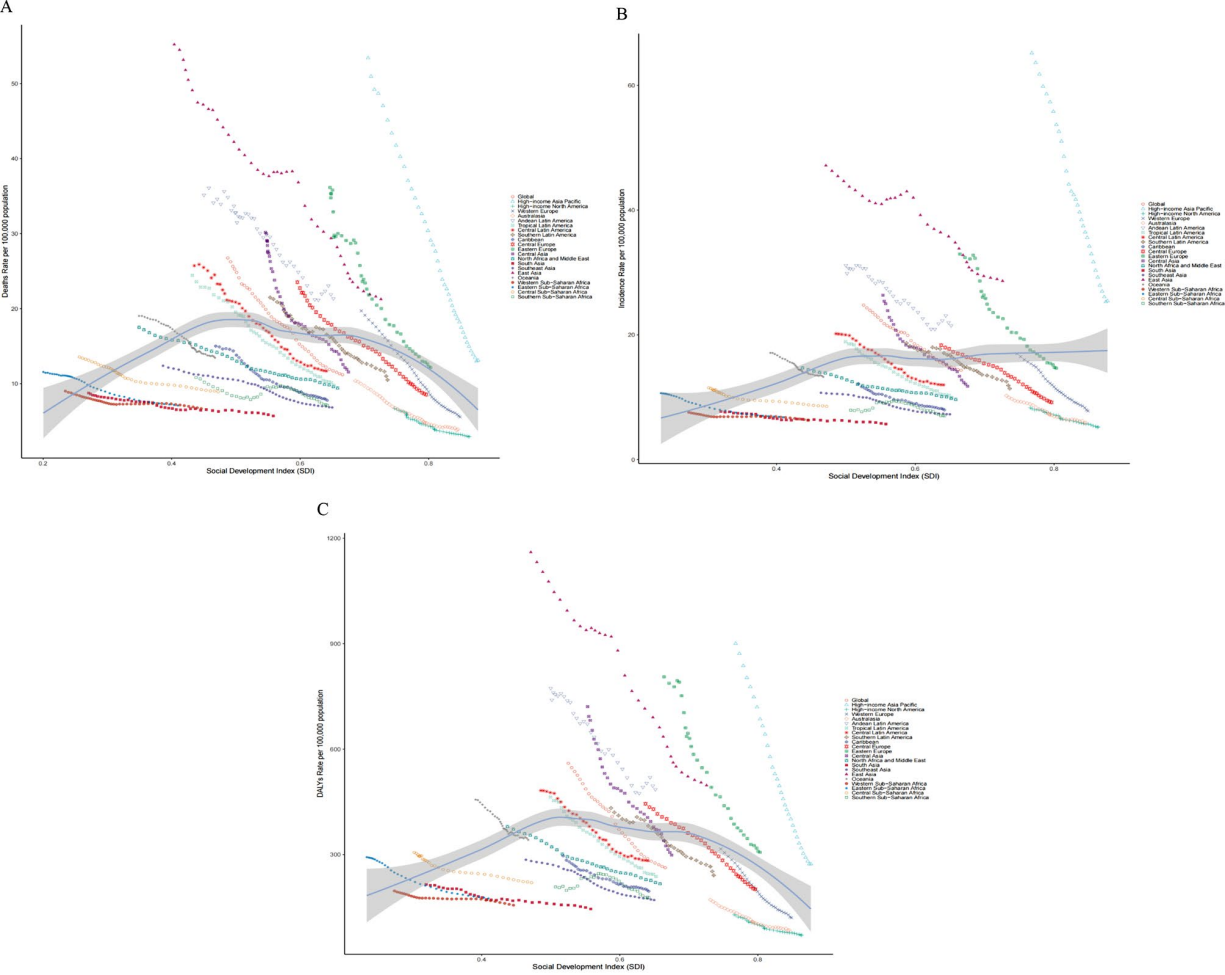
The trend in age-standardized death (**A**), incidence (**B**), and DALYs (**C**) rates of gastric cancer in 21 GBD regions by SDI, 1990–2021. For each region, points from left to right depict estimates from each year from 1990 to 2021. The blue line represents the average expected relationship between SDI and burden estimates rates for gastric cancer based on values from each geographical region over the 1990–2021 estimation period. *DALYs* disability-adjusted life-years, *SDI* socio-demographic index

**Fig. 4:**
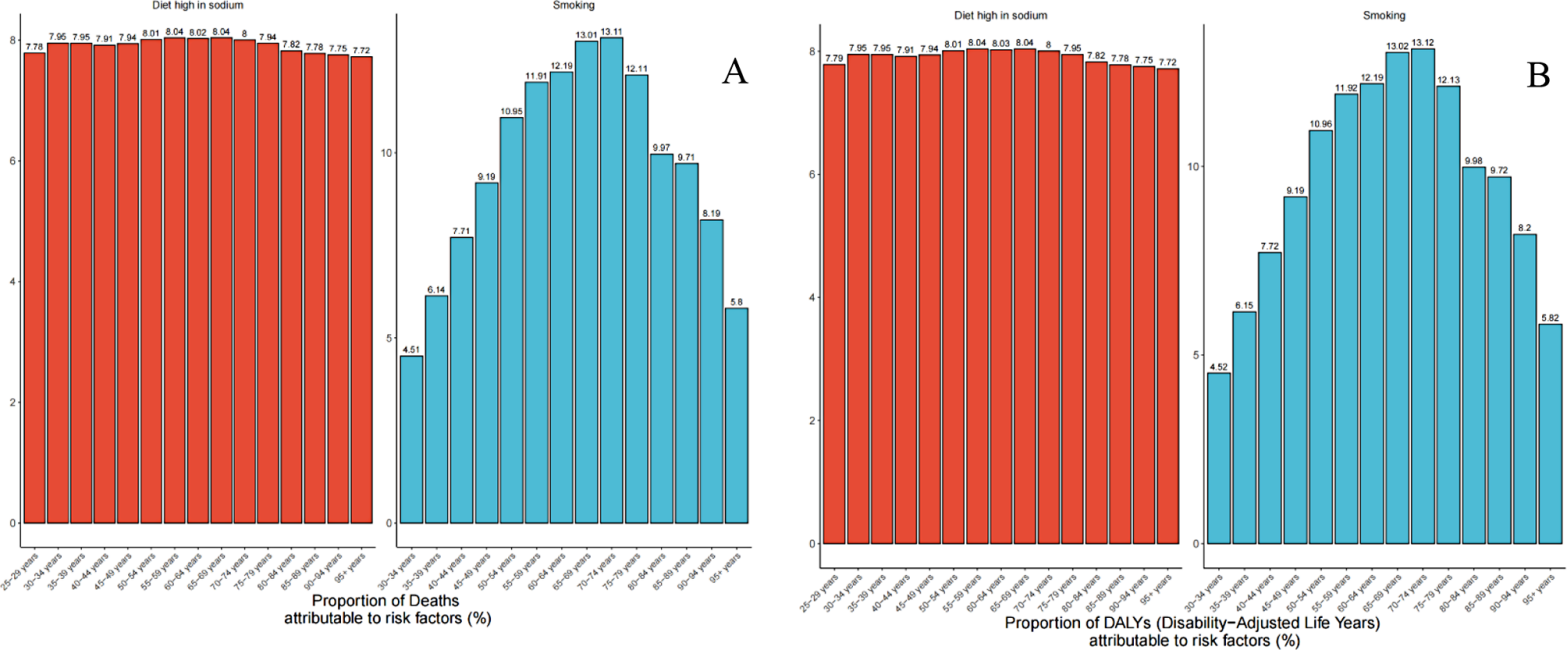
Proportion of gastric cancer death and DALYs risk factors by age in 2021

### GC incidence and MRs vis-à-vis SDI

Figure 3 depicts the long-term trends in the ASDRs, ASIRs, and ASRs for GC across SDI quintile categories by geographical area from 1990 to 2021, together with the projected rates derived exclusively from the SDI measurements of worldwide regions. Significant variations in the impact of SDI across regions were observed. A general downward trajectory was identified in most regions. The anticipated ASIRs, ASDRs, and ASRs showed a reduction with increasing SDI values. Notably, the ASDR in high-income Asia Pacific and Central Asia significantly declined as SDI values increased (Fig. 3A).

As the SDI value increased, a pronounced reduction in the ASIR was noted in high-income Asia Pacific and Eastern Europe, while a more gradual diminish occurred in South Asia and Western Sub-Saharan Africa. In East Asia, the ASIR initially declined, followed by a slow increase, peaking at an SDI of 0.6, and then experienced a marked reduction as the SDI value continued to rise (Fig. 3B). The correlation between SDI and the ASR mirrored that with the ASDR (Fig. 3C).

### GC risk factors

In 2021, the highest proportions of ASDRs and ASRs for GC linked to smoking were observed in the 70–74 age cohort, while the greatest attribution to high-sodium diets occurred in the 55–59 and 65–69 age cohorts (Fig. 4).

**Fig. 5:**
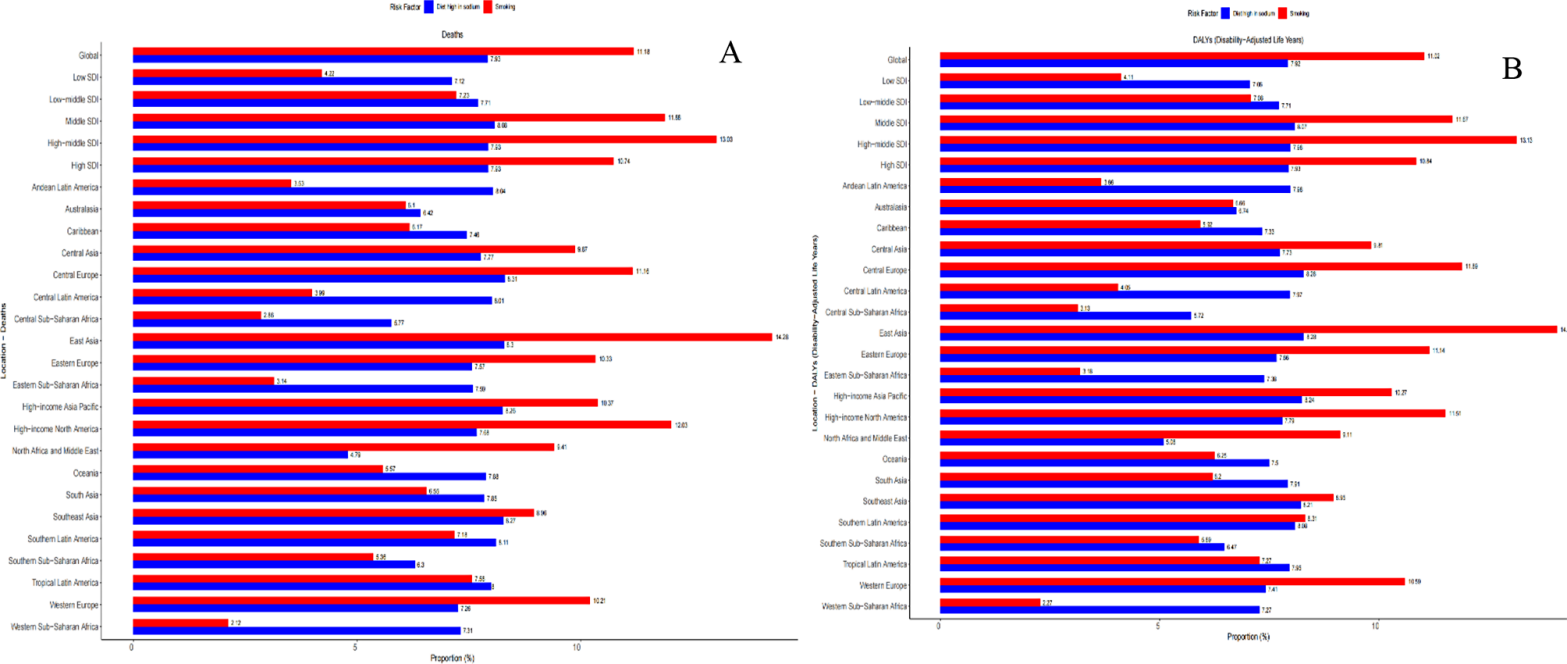
Proportion of gastric cancer deaths and DALYs attributable to risk factors, for global and regions in 2021

From 1990 to 2021, global and regional trends in ASDRs and ASRs linked to high-sodium diets and smoking exhibited notable variation. Worldwide, 7.93% of the ASDRs and 11.18% of the ASRs were attributed to high-sodium diets and smoking, respectively. Furthermore, 7.92% and 11.02% of the ASRs were ascribed to these factors, respectively (Fig. 5). Smoking had a greater impact on ASRs in middle SDI, high-middle SDI, and high SDI regions than low SDI and low-middle SDI regions, where high-sodium diets played a more prominent role. Although smoking represented a smaller proportion of ASRs on a global scale, it accounted for over 12% of ASRs in East Asia and high-income North America for GC.

**Fig. 6:**
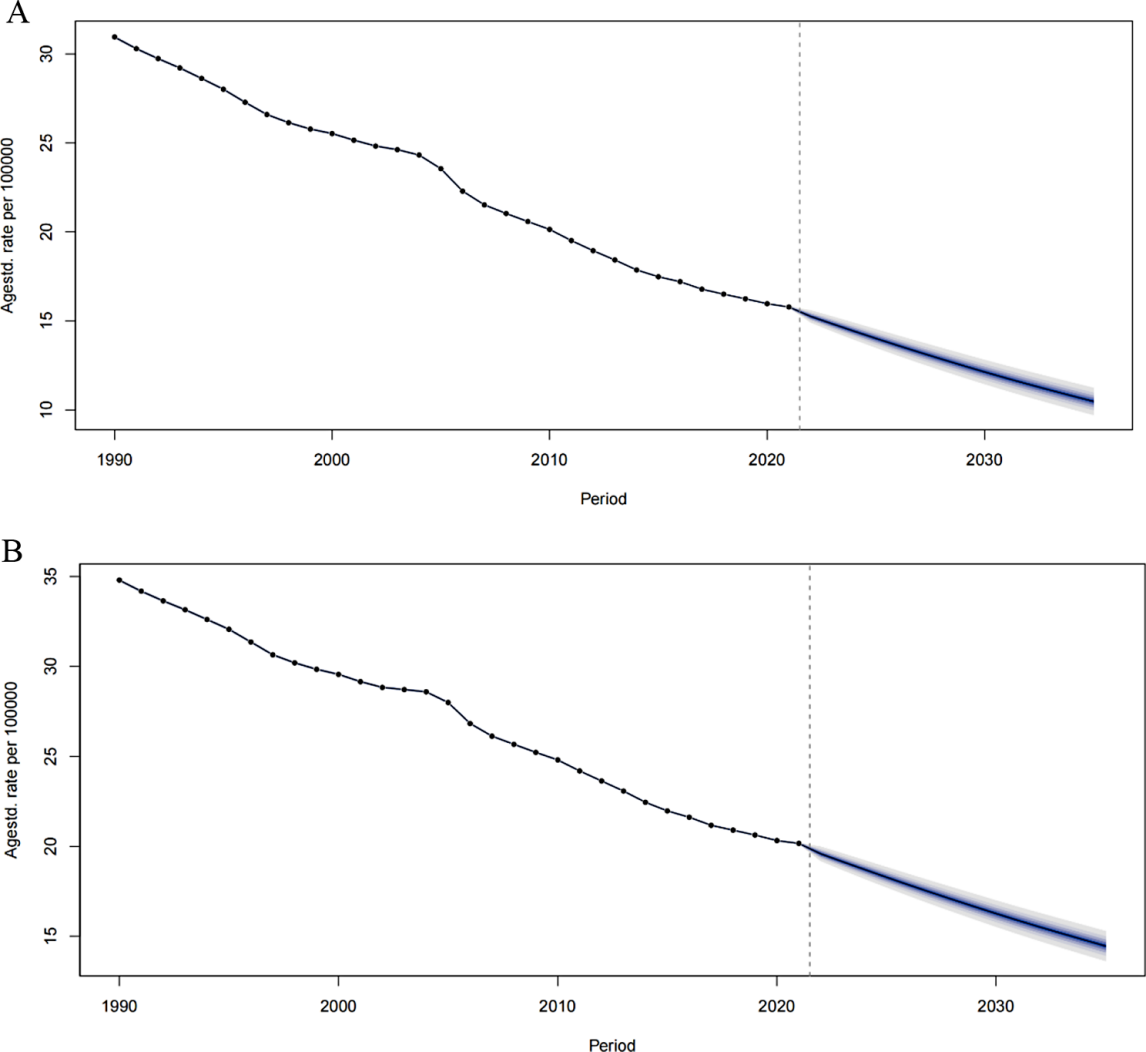
The change trends of the cancer-related disease burden by age from 1990 to 2035. **A**. The ASDR of gastric cancer. **B**. The ASIR of gastric cance

In 2021, East Asia exhibited the most incredible ASDR connected to tobacco use, with high-income North America ranking second, while Western Sub-Saharan Africa demonstrated the minimum rate (Fig. 5A). Central Europe registered the most elevated ASDR related to excessive sodium consumption. Moreover, Central Europe showed the peak ASR concerning high-sodium dietary patterns, whereas North Africa and the Middle East displayed the lowest figures (Fig. 5B).

### Predictions of GC from 2022 to 2032

The BAPC model was utilized to predict the ASIR and ASDR of GC across various age cohorts from 2022 to 2035. A continuous decline in both ASDR and ASIR is anticipated during this period (Fig. 6). Projections suggest that the ASDR for GC across different age cohorts will either stabilize or continue to decline, mirroring the decreasing trend seen between 1990 and 2021. Notably, the ASDR for the 45–49, 50–54, and 70–74 age cohorts is expected to stabilize or plateau, contrasting with the significant reductions seen from 1990 to 2021. For the 90–94 and 95 + age cohorts, the ASDR did not exhibit substantial declines between 1990 and 2021; however, a significant decrease is predicted for these cohorts in the upcoming years (Additional file). Furthermore, the ASIR for the 70–74 and 75–79 age cohorts is also expected to experience a notable decline, in line with the pattern observed between 1990 and 2021 (Additional file).

## Discussion

GC has evolved into a crucial worldwide health issue, marked by elevated morbidity rates and MRs, necessitating heightened attention and intervention efforts [19]. This investigation provides the most recent temporal and geographical insights into the global burden of GC, examining regional and country-specific patterns from 1990 to 2021, utilizing GBD 2021 study information. The findings revealed that although absolute numbers of GC incidents, DALYs, and fatalities showed an upward trend between 1990 and 2021, the ASIRs, ASRs, and ASDRs demonstrated downward movement during this timeframe. The worldwide ASDR and ASIR for GC reached 11.2 and 14.33 per 100,000 person-years, respectively, in 2021, showing a reduction from 2019 figures (ASDR, 12; ASIR, 16 per 100,000 person-years) [3]. While global ASIR and ASDR values continue declining, demographic aging, lifestyle modifications, and expanding population numbers might contribute to higher absolute GC occurrence and mortality figures [20].

Additionally, an unusual trend was observed during the COVID-19 pandemic, from 2019 to 2021, with both global mortality and incidence rates exhibiting an upward trend, particularly in East Asian countries. This reversal in the downward trend of GC burden was attributable to the pandemic’s impact on healthcare systems. The COVID-19 pandemic disrupted healthcare services globally, precipitating delays in cancer screening, diagnosis, and treatment, which likely contributed to the observed increase in GC cases and deaths during this period. In East Asia, where GC is already a significant health concern, these disruptions may have exacerbated the disease burden, underscoring high-incidence regions’ vulnerability to healthcare system interruptions.

The burden of GC continues to exhibit substantial geographical variation across regions globally. However, the incidence rates have consistently declined in most nations and are projected to persist in this trend moving forward [21]. It was observed that the highest SDI quintile demonstrated the lowest ASDR and a reduced ASIR for GC. The substantial decline in ASIR primarily stemmed from modifications in daily habits and sanitation protocols, encompassing reduced intake of salt-preserved and smoked food items, coupled with diminished Hp infection rates [22, 23]. In contrast, regions in the lowest SDI category exhibited relatively minimal GC impact, demonstrating decreases in both death rates and disease occurrence. This variation can be attributed to the underestimation of cancer statistics and mortality data in these areas, resulting from diagnostic errors, undetected cases, limited medical facilities, and inadequate cancer monitoring mechanisms, further complicated by disparate resource allocation [24].

The peak occurrence of GC was documented in East Asia, confirming earlier research findings [25]. Andean Latin America demonstrated the most substantial GC mortality figures, with East Asia ranking second. High-income North America and Australasia displayed the minimum ASDRs, supporting prior scientific observations [26].

The global implementation of prevention, screening, and treatment programs has contributed to a steady decline in both occurrence and MRs of GC across most countries over recent decades [27, 28].

On a nationwide scale, Mongolia, Afghanistan, and Bolivia exhibited the most elevated ASIRs and ASDRs for GC. Subsequently, China and the Republic of Korea demonstrated the next substantial ASIRs. Within the Asian continent, the five nations recording the maximum ASIRs comprised Mongolia (36.83), Afghanistan (32.93), China (29.05), the Republic of Korea (25.76), and Japan (25.54). These findings differ from earlier research outcomes. Based on data from GLOBOCAN 2020 (https://gco.iarc.fr/), the five Asian nations displaying the peak ASIRs were documented as Mongolia (32.5), Japan (31.6), South Korea (27.9), Tajikistan (23.4), and China (20.6) [4, 29]. Although GC ASIRs have typically shown a downward pattern across numerous countries, Mongolia and China have experienced an upward trajectory in occurrence rates. Mongolia’s elevated incidence and MRs stem largely from limited endoscopic resources and specialists [30]. GC’s initial manifestations tend to be inconspicuous, resulting in most patients being diagnosed at advanced metastatic stages [31]. Limited screening awareness has contributed to low detection rates of early-stage cancer, adversely affecting both prognosis and the overall burden on patients [32]. Over recent decades, a consistent decline in GC incidence has been recorded in China. With the growing availability of gastrointestinal endoscopy, China has implemented several protocols focused on enhancing screening, early detection, and treatment strategies for GC. As a result, the proportion of early-stage GC cases has risen lately, with most instances now being identified through early screening [33]. Notably, Japan has observed a significant reduction in GC burden, while Western countries, encompassing the United States, have reported relatively low GC MRs. The five-year survival rates for GC have shown improvement, primarily attributed to advancements in diagnostic techniques and treatment protocols, particularly early identification facilitated by endoscopic and/or radiological procedures incorporated into countrywide screening initiatives [34]. For instance, research indicated that the decline in GC MRs in the United States could be linked to enhanced treatment approaches and better survival outcomes, supported by the development of advanced surgical procedures and preoperative chemotherapy [35]. Furthermore, the investigation highlighted that European countries exhibited diminished ASIRs and ASDRs for GC globally, likely owing to substantial improvements in living conditions, which have contributed to increased survival rates and reduced morbidity and mortality through early detection, improved healthcare access, and better treatment methods [36–38].

The discrepancies in the ASDRs and ASIRs of GC across various regions could be linked to the heterogeneous presence of RFs within diverse populations. The findings of this study suggest that a high-sodium diet markedly contributes to the burden of GC. Elevated salt concentrations are known to directly impair the gastric mucosa, inducing hyperplasia of the pit epithelium, thereby enhancing the probability of endogenous mutations [39]. Furthermore, investigation indicates that high sodium consumption accelerates intestinal metaplasia progression, potentially leading to the development of early-stage GC [40]. Previous research has suggested that excessive sodium consumption may modify the thickness of the defensive mucus layer and facilitate the proliferation of Hp, a well-established RF for GC [41]. Although people of every age group face risks from excessive sodium consumption, subtle differences in the ASDRs and ASIRs of GC are observed among various age cohorts exposed to high sodium levels. Owing to the progressive decline in bodily functions and immune system efficiency, the elderly population is particularly vulnerable to the harmful effects of a high-sodium diet, with the ASDR and ASIR of GC among this cohort frequently showing significant elevations when exposed to elevated sodium consumption.

Additionally, significant geographical disparities in the ASDRs and ASIRs of GC, attributed to smoking, were observed globally. Smoking continues to be recognized as a prominent RF for GC. Following the implementation and endorsement of the WHO Framework Convention on Tobacco Control in 2005, smoking prevalence has consistently declined throughout numerous nations and territories [42], thereby accounting for the reduced impact of smoking on the global GC burden over the last ten years. Nevertheless, in East Asia, the contribution of smoking to the ASRs and ASDRs of GC remains notably high, with 14.05% of ASRs and 14.28% of ASDRs attributable to smoking—substantially exceeding the levels observed in other regions.

Guidelines for promoting healthy lifestyles consistently advocate for the reduction of tobacco exposure [43]. In high-risk East Asian countries, the burden of GC can be mitigated through the implementation of comprehensive strategies—these include intensifying tobacco control measures, enacting legislation to restrict tobacco product manufacturing and distribution, enhancing community knowledge about smoking dangers, promoting the embrace of more beneficial lifestyle choices, and cultivating cross-border partnerships to exchange scientific findings and effective protocols.

A significant limitation of the present study lies in the examination of only two RFs for GC. Other critical RFs, such as Hp infection, also contribute to the GC burden. GC is categorized into two forms: cardia and non-cardia. The latter is primarily linked to Hp infection, which may account for the observed changes in global GC incidence [44]. However, due to the paucity of data in the GBD database, Hp infection was not included in this analysis.

## Conclusion

GC continues to impose a substantial global burden, affecting various regions and populations. This study has demonstrated a notable reduction in both incidence and MRs of GC, which is especially evident among females and individuals in the high SDI quintile. While mortality and DALYs associated with high-sodium diets and smoking have shown overall decreases, marked disparities remain between different age cohorts and geographical regions. The predictive models developed in this study suggest that the worldwide GC burden will improve in the coming decade. Nevertheless, implementing robust preventive measures and therapeutic approaches—encompassing enhanced smoking control and higher intake of fresh fruits and vegetables—are essential for reducing GC occurrence.

## Electronic supplementary material

Below is the link to the electronic supplementary material.

Supplementary Material 1

Supplementary Material 2

## Data Availability

GBD study 2021 data resources were available online from the Global Health Data Exchange (GHDx) query tool (http://ghdx.healthdata.org/gbd-results-tool). All data generated or analyzed during this study are included in this article and supplementary material.
